# The Role of *VEGFA*, *COX2*, *HUR* and *CUGBP2* in Predicting the Response to Neoadjuvant Therapy in Rectal Cancer Patients

**DOI:** 10.3390/medicina56040192

**Published:** 2020-04-22

**Authors:** Henrikas Pauzas, Ugne Gyvyte, Tadas Latkauskas, Laura Kairevice, Paulius Lizdenis, Saulius Svagzdys, Erika Birgiolaite, Irma Kuliaviene, Juozas Kupcinskas, Algimantas Tamelis

**Affiliations:** 1 Department of Surgery, Academy of Medicine, Lithuanian University of Health Sciences, LT-50161 Kaunas, Lithuania; henrikas.pauzas@lsmuni.lt (H.P.); tadas.latkauskas@lsmuni.lt (T.L.); paulius.lizdenis@lsmuni.lt (P.L.); saulius.svagzdys@lsmuni.lt (S.S.); erikabirgiolaite@yahoo.com (E.B.); 2 Institute for Digestive Research, Academy of Medicine, Lithuanian University of Health Sciences, LT-50161 Kaunas, Lithuania; ugne.gyvyte@lsmuni.lt (U.G.); juozas.kupcinskas@lsmuni.lt (J.K.); 3 Department of Oncology and Hematology, Academy of Medicine, Lithuanian University of Health Sciences, LT-50161 Kaunas, Lithuania; laura.kairevice@lsmuni.lt; 4 Department of Gastroenterology, Academy of Medicine, Lithuanian University of Health Sciences, LT-50161 Kaunas, Lithuania; irma.kuliaviene@lsmuni.lt

**Keywords:** rectal cancer, neoadjuvant therapy, VEGFA, COX2, HUR, CUGBP2

## Abstract

*Background and objectives*: The effectiveness of neoadjuvant therapy, which is commonly used for stage II-III rectal cancer (RC) treatment, is limited. Genes associated with the pathogenesis of RC could determine response to this treatment. Therefore, the aim of this study was to investigate the potential predictive value of *VEGFA*, *COX2*, *HUR* and *CUGBP2* genes and the associations between post-treatment changes in gene expression and the efficacy of neoadjuvant therapy. *Materials and Methods*: Biopsies from RC and healthy rectal tissue of 28 RC patients were collected before neoadjuvant therapy and 6-8 weeks after neoadjuvant therapy. The expression levels of *VEGFA*, *COX2*, *HUR*, *CUGBP2* genes were evaluated using a quantitative real-time polymerase chain reaction. *Results*: The results reveal a significantly higher expression of *VEGFA*, *COX2* and *HUR* mRNA in RC tissue compared to healthy rectal tissue (*p* < 0.05), and elevated *VEGFA* gene expression in pre-treatment tissues was associated with a better response to neoadjuvant therapy based on T-stage downstaging (*p* < 0.05). The expression of *VEGFA*, *HUR* and *CUGBP2* genes significantly decreased after neoadjuvant therapy (*p* < 0.05). Responders to treatment demonstrated a significantly stronger decrease of *VEGFA* and *COX2* expression after neoadjuvant therapy than non-responders (*p* < 0.05). *Conclusions*: The findings of this study suggest that the pre-treatment *VEGFA* gene expression might have predictive value for the response to neoadjuvant therapy, while the post-treatment decrease in *VEGFA* and *COX2* gene expression could indicate the effectiveness of neoadjuvant therapy in RC patients.

## 1. Introduction

Colorectal cancer (CRC) screening programs have huge potential in reducing the burden of CRC in countries that have well-running screening platforms [1]. Nevertheless, current estimates of the worldwide incidence and mortality from rectal cancer (RC) obligate us to look for more precise diagnostics and better treatment options [2]. Neoadjuvant therapy with delayed surgery is the most common regimen reducing the local recurrence rate for patients with resectable rectal cancer. Tumor downstaging and the pathological complete response has been associated with the effectiveness of neoadjuvant therapy and could be prognostic factors to predict individual clinical outcome [3,4]. However, the effectiveness of neoadjuvant therapy is limited, and the downstaging rate ranges between 40% and 60% [3,5,6,7,8]. 

The pathogenesis of CRC is complex and may involve multiple genetic [9,10,11,12], epigenetic [13,14] and environmental factors [15] that may contribute to tumor development. Recent studies suggested that genes associated with the pathogenesis of RC, such as vascular endothelial growth factor A (*VEGFA*), cyclooxygenase 2 (*COX2*), human antigen R (*HUR*) and CUG triplet repeat RNA binding protein (*CUGBP2*), and their products could predict the response to neoadjuvant therapy and, therefore, improve clinical care by selecting an optimal treatment for RC patients, or be prognostic factors in colorectal cancer (CRC) [16,17,18,19,20,21,22,23,24,25,26,27,28,29,30,31,32,33]. The overexpression of *COX2* has been observed in multiple cancers, including CRC, and can induce the expression of *VEGF*, leading to increased angiogenesis and tumor progression [34,35], while AU-rich element-binding (ARE) proteins HUR and CUGBP2 have been shown to stabilize and modulate *COX2* expression [36]. 

In this study, we aimed to evaluate the associations between expression levels of *VEGFA*, *COX2*, *HUR* and *CUGBP2* genes and the response to neoadjuvant therapy in RC patients. 

## 2. Materials and Methods

### 2.1. Study Population and Samples

Research of *VEGFA*, *COX2*, *HUR* and *CUGBP2* gene expression in rectal cancer was the experimental part of a prospective randomized trial “Preoperative conventional chemoradiotherapy versus short-course radiotherapy with delayed surgery for rectal cancer” [37]. The study was approved by the Kaunas Regional Committee of Ethics of Biomedical Research (Protocol No. P2-137/2006, 8 December 2011). All patients have signed an informed consent form to participate in the study. The inclusion, exclusion criteria and initial results of the aforementioned prospective trial have been previously reported [38,39]. In short, patients under 80 years old with histologically confirmed stage II and III rectal cancer, located less than 15 cm from the anal verge, with no other cancer during the previous five years and normal cardiovascular, pulmonary, hepatic and renal functions, were included in the study. Initially, it was planned to collect RC and healthy rectal tissue samples of 50 RC patients for the gene expression analysis during the years 2011–2013; however, due to the progression of the disease, the cancellation of the surgery, an insufficient amount of tissue sample or an insufficient amount and poor quality of RNA, and the inability to take the samples repeatedly, 27 RC patients were finally investigated in the study. The characteristics of patients included in the study are provided in Table 1. The downstaging of the tumor was evaluated, comparing changes in the clinical and pathological stage. Clinical T and N stages were assessed using endorectal ultrasound and magnetic resonance imaging before the neoadjuvant therapy and re-evaluated after radiotherapy, before the surgery. The post-surgical pathological stage was determined based on the pathologist’s conclusions. The pathological “T/N-stage downstaging” (comparing post-surgical ypT/ypN to the pre-treatment cT/cN) has been considered when clinical stage cII-III has become a pathological p0-I stage (according to the American Joint Committee on Cancer (AJCC) staging system) after neoadjuvant therapy. “T-stage downstaging” has been considered when the pathological T-stage decreased after neoadjuvant therapy comparing to the clinical T-stage (comparing post-treatment ypT to the pre-treatment cT). A summary of patient distribution according to AJCC and TNM staging systems before and after neoadjuvant therapy is provided in the Table S1 of the Supplementary Materials.

Tissue samples were taken during the endoscopic examination before neoadjuvant therapy (*n* = 27) and repeatedly taken 6–8 weeks after neoadjuvant therapy (conventional chemoradiotherapy (CRT; 50 Gy in total administered during a period of 5 weeks, 2 Gy per fraction and two cycles of 5-FU/Leucovorin, 400 mg/m^2^ of 5-fluorouracil i/v in combination with leucovorin 20 mg/m^2^ i/v for 1–4 days on the first and on the fifth week) or short-term radiotherapy (RT; 5 fractions of radiotherapy, 5 Gy per fraction, administered each day for 5 days, a dose of 25 Gy in total)) on surgery day after tumor removal (*n* = 12). To stabilize RNA, biopsies were treated with “RNA later^®^ Solution” and frozen to −80 °C after 24 h.

### 2.2. RNA Isolation

Up to 50 mg of tissue was used for RNA isolation using a “Direct-zol^TM^ RNA MiniPrep” (Zymo Research, Irvine, CA, USA) Kit. RNA concentration and purity were evaluated by spectrophotometer NanoDrop 2000 (>150 ng/µL RNA concentration and A260/A280 purity value 1.8–2.0 were required for further research). Extracted RNA was further stored at −80 °C.

### 2.3. Reverse Transcription and qPCR Analysis

Isolated RNA was used for complementary DNA (cDNA) synthesis by using a SuperScript^®^ VILO^TM^ cDNA Synthesis Kit (Invitrogen^TM^, Carlsbad, CA, USA) according to the manufacturer’s protocol. In order to detect the expression of *VEGFA*, *COX2*, *HUR* and *CUGBP2* genes, the resulting cDNA was subjected to the quantitative real-time PCR (qPCR) using primers and probes from TaqMan^®^ Gene Expression Assays (Hs0090005_m1 *VEGFA*, Hs00153733_m1 *PTGS* (*COX2*), Hs00171309_m1 *ELAVL1* (*HUR*), Hs00272516_m1 *CELF2* (*CUGBP2*)) together with TaqMan^®^ Universal PCR Mastermix II (no UNG) on the 7500 Fast Real-Time PCR System (Applied Biosystems, Foster City, CA, USA), according to the manufacturer’s recommendations. The expression data were normalized to the expression levels of *ACTB* reference gene.

### 2.4. Statistical Analysis

qPCR data were analyzed using the comparative C_T_ method. Differences in gene expression between investigated groups were evaluated using a Wilcoxon signed-rank test (for dependent samples, including RC vs. healthy tissue, before vs. after therapy) or a Wilcoxon rank-sum test (for independent samples, including downstaging vs. non-downstaging groups) for non-parametric data. Pearson’s correlation coefficients were calculated to determine the correlations between gene expression levels. Differences between the investigated groups were considered significant when *p* < 0.05. All statistical analyses were performed using the statistical computing environment R (version 3.5.2).

## 3. Results

### 3.1. Expression of VEGFA, COX2 and HUR is Altered in Rectal Cancer Tissue

**Figure 1:**
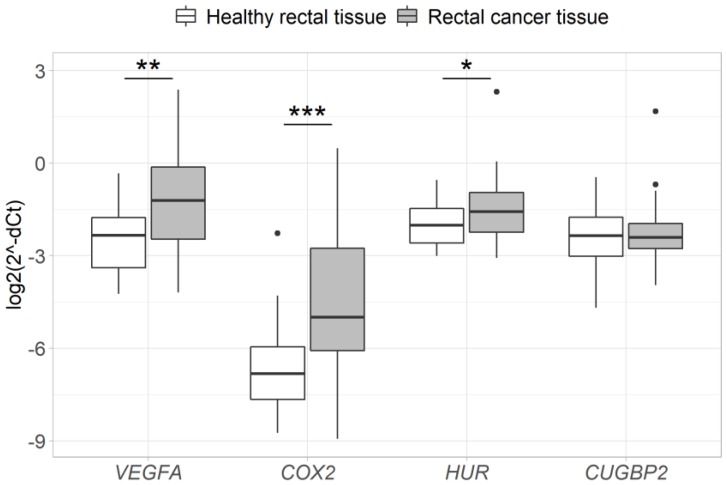
Expression of *VEGFA*, *COX2*, *HUR* and *CUGBP2* genes in RC vs. healthy rectal tissue. * - *p* < 0.05, ** - *p* < 0.005, *** - *p* < 0.0005. Gene expression was normalized to the expression levels of the *ACTB* reference gene.

**Figure 2:**
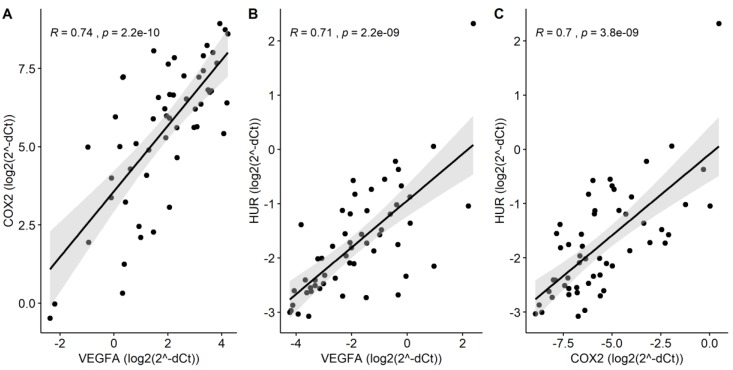
A significant correlation between the expression levels of RC-deregulated genes. A high positive correlation between the expression of (**A**) *COX2* and *VEGFA*; (**B**) *HUR* and *VEGFA*; (**C**) *HUR* and *COX2* genes. R—Pearson’s correlation coefficient; correlation is significant when *p <* 0.05.

Analysis of *VEGFA*, *COX2*, *HUR* and *CUGBP2* gene expression in the samples taken before neoadjuvant therapy (*n* = 27) revealed a significant upregulation of *VEGFA*, *COX2*, and *HUR* mRNA in RC tissue compared to healthy rectal mucosa (2.4-fold, *p* = 9.2 × 10^−4^; 5-fold, *p* = 7.3 × 10^−5^; 1.4-fold, *p* = 0.02, respectively), while no difference was observed in the expression levels of *CUGBP2* mRNA (*p* > 0.05) (Figure 1). In addition, high positive correlations were observed between the expression levels of *VEGFA* and *COX2* (R = 0.74, *p* = 2.2 × 10^−10^) or *HUR* (R = 0.71, *p* = 2.2 × 10^−9^), as well as between *COX2* and *HUR* (R = 0.7, *p* = 3.8 × 10^−9^) genes (Figure 2).

### 3.2. Pre-Treatment Expression of VEGFA Is Associated with a Response to Neoadjuvant Therapy

**Figure 3:**
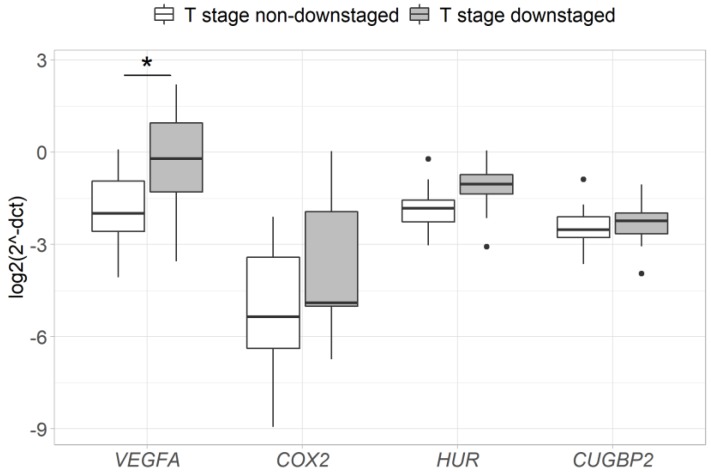
Pre-treatment levels of *VEGFA*, *COX2*, *HUR* and *CUGBP2* gene expression compared between the groups of RC patients with and without T-stage downstaging (following neoadjuvant therapy). *—*p* < 0.05. Gene expression was normalized to the expression levels of the *ACTB* reference gene.

**Figure 4:**
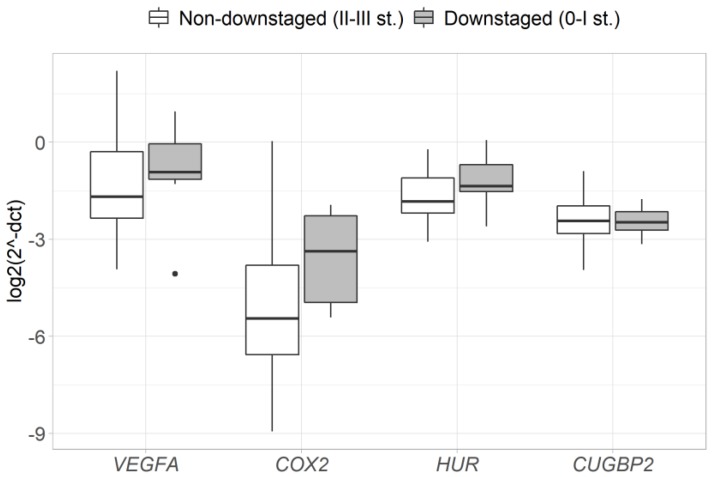
Pre-treatment levels of *VEGFA*, *COX2*, *HUR* and *CUGBP2* gene expression compared between the groups of RC patients with and without pathological T/N-stage downstaging (cII-III to ypI-II) (following neoadjuvant therapy). Gene expression was normalized to the expression levels of the *ACTB* reference gene.

In order to evaluate the association between the pre-treatment expression and response to neoadjuvant therapy, differences of gene expression between the groups of responders and non-responders (based on pathological T/N-stage and T-stage downstaging) were assessed. The results reveal that tumors in a T-stage downstaging group had a significantly higher expression of the *VEGFA* gene before the neoadjuvant therapy (2.9-fold, *p* = 0.028) (Figure 3), while no difference in gene expression was observed between pathological T/N-stage downstaging and non-downstaging groups (Figure 4). 

### 3.3. Changes in VEGFA and COX2 Expression during Neoadjuvant Therapy is Associated with a Response to Treatment

**Figure 5:**
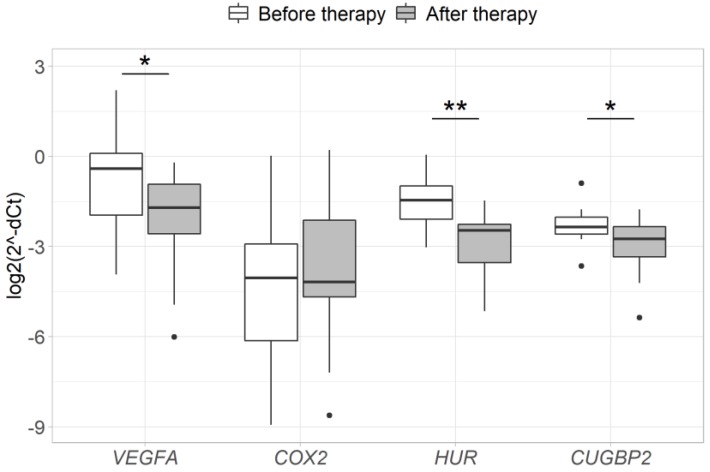
Expression of *VEGFA*, *COX2*, *HUR* and *CUGBP2* genes in RC tissue before vs. after neoadjuvant therapy. *- *p* < 0.05, ** - *p* < 0.005, *** - *p* < 0.0005. Gene expression was normalized to the expression levels of the *ACTB* reference gene.

**Figure 6:**
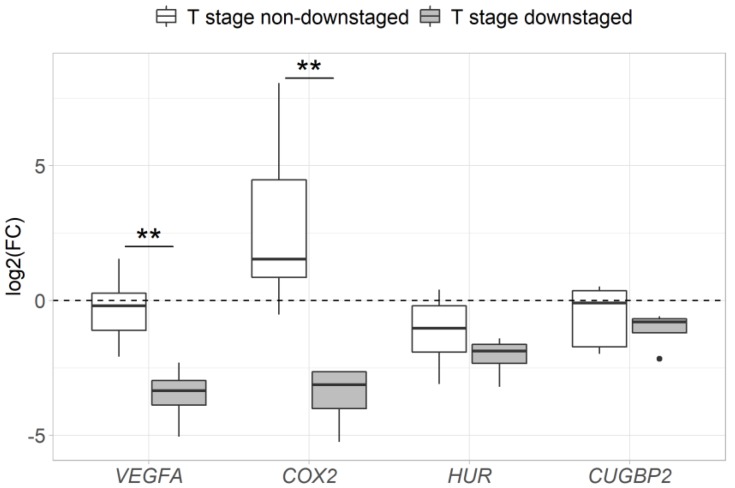
Differences in fold changes of gene expression after neoadjuvant therapy compared between the groups of responders and non-responders (based on T-stage downstaging).

Changes in the expression of *VEGFA*, *COX2*, *HUR* and *CUGBP2* genes after neoadjuvant therapy were successfully evaluated in the group of 12 patients. Due to the small sample size, CRT- and RT- treated patients were analyzed as a single neoadjuvant therapy group. Analysis revealed a significant decrease in *VEGFA*, *HUR* and *CUGBP2* gene expression after neoadjuvant therapy compared to the expression levels before the treatment (2.6-fold, *p* = 0.034; 2.8-fold, *p* = 0.002; 1.7-fold, *p* = 0.034, respectively). No significant changes in the expression of the *COX2* gene were observed after neoadjuvant therapy (*p* > 0.05) (Figure 5). Furthermore, the group of T-stage responders demonstrated a more prominent *VEGFA* and *COX2* expression decrease after neoadjuvant therapy than the group of T-stage non-responders (*p* < 0.05) (Figure 6). 

## 4. Discussion

In the present study, we analyzed the expression of *VEGFA*, *COX2*, *HUR* and *CUGBP2* genes and their alterations in response to neoadjuvant therapy in the tissues of RC and healthy rectal mucosa. We further evaluated the associations between gene expression and patient response to neoadjuvant treatment. The major findings of our study indicate the possible predictive value of pre-treatment *VEGFA* mRNA expression for the response to neoadjuvant therapy, while post-treatment changes in the expression of *VEGFA* and *COX2* mRNA may have potential, indicating the effectiveness of neoadjuvant therapy in RC patients.

Higher levels of *VEGFA*, *COX2* and *HUR* mRNA were detected in RC tissue compared to the healthy rectal mucosa. *VEGFA* gene encodes a well-studied pro-angiogenic factor that induces endothelial cell proliferation, migration, survival, and therefore contributes to tumor angiogenesis [40]. COX2 is an enzyme induced by growth factors, inflammatory mediators, and tumor promoters [34]. There is sufficient data confirming positive correlations between the amount of COX2 protein in cancerous tissue and staging, metastasis and survival rate in a variety of tumors, including CRC [34,41,42,43]. It has been shown that the overexpression of COX2 and its enzymatic product prostaglandin E2 (PGE2) are related to the increased amount of VEGFA, which promotes the formation of various small vessels in tumors [34,44]. Although there are a lot of data on COX2 and VEGFA protein expression in the tissues of CRC, there are only a few publications on their mRNA expression. Our results are consistent with the prior data found in the literature describing the upregulation of *COX2* and *VEGFA* genes in the cancerous tissues of colon and rectum compared to healthy tissues [17,22,23,45,46] and confirm that changes in the expression of these genes happen already at the mRNA level during pathological processes. Moreover, a positive correlation between the expression levels of *COX2* and *VEGFA* mRNA was found, supporting the previously described interaction between COX2 and VEGFA [34,47].

The products of *HUR* and *CUGBP2* genes take part in the regulation of *VEGFA* and *COX2* translation to protein, selectively attaching to ARE sequences in their mRNA [26,36,48]. However, the mechanisms of gene expression regulation by these RNA-binding proteins are not entirely clear. It has been proven that HUR protein is overexpressed in CRC, is associated with the activation of *COX2* and *VEGFA* gene expression in tumor endothelial cells and, therefore, is an important factor activating angiogenesis in tumor cells and plays an important role in cancer development and progression by targeting the mRNAs of proto-oncogenes, cytokines, growth and invasion factors [23,24,25,26,48,49]. In this study, we found a significant increase in *HUR* mRNA expression in RC tissue compared to the surrounding healthy tissue and a high positive correlation between expression levels of *HUR* and *COX2* or *VEGFA*, which confirmed the results of a study performed by Young et al. [23]. Contrarily, CUGBP2 has been shown to be downregulated in colon cancer, inhibit *COX2* mRNA translation to protein and prevent cancer development [50,51]. However, no differences were observed in *CUGBP2* gene expression comparing healthy and cancerous rectal tissues in this study. Few studies, evaluating changes in *CUGBP2* gene expression, have been reported, suggesting that the *CUGBP2* gene expression changes only when affected by radiation [29].

It has been previously shown that *VEGFA*, *COX2* and *HUR* genes could be promising predictive markers for the response to neoadjuvant therapy [49,52,53,54]. We found a higher pre-treatment expression of *VEGFA* mRNA in patients with a T-stage downstaging, indicating a higher efficiency of the neoadjuvant treatment. These results are in line with the data published by Hur and colleagues, where RC patients with high VEGFA expression levels exhibited a significantly greater pathological complete response rate [55]. These results could be explained by the increased tumor vascularization caused by the higher expression of VEGFA and, therefore, a better supply of oxygen required for the cytotoxic activities of CRT and RT [56]. However, other studies present inconsistent results where greater VEGFA expression has been found in non-responsive tumors or was unrelated to the response to preoperative RT or CRT [57,58]. Although the pre-treatment gene expression difference between the T/N-stage responders and non-responders were in the same direction as between T-stage responders and non-responders, the difference of *VEGFA* expression was not significant in this group. This could be due to a lower number of patients with T/N-stage downstaging and the analysis in a larger group of patients would be useful to draw more specific conclusions. 

Analysis of *VEGFA*, *COX2*, *HUR* and *CUGBP2* gene expression in RC tissue after neoadjuvant therapy revealed a significantly decreased expression of *VEGFA*, *HUR* and *CUGBP2* mRNA, while changes in *COX2* mRNA expression were not significant. Our results could be compared to the study published by Murmu et al., where the changes in *COX2*, *CUGBP2*, *HUR* mRNA and protein expression have been analyzed in mice small intestine mucosa after radiotherapy [28]. An increase in *CUGBP2* mRNA expression in mice intestinal mucosa has been observed 6 h after radiotherapy and has been associated with an increased apoptosis of cancer cells and better effectiveness of the treatment. However, a decrease in the *CUGBP2* gene expression and an increase in *COX2* and *HUR* gene expression have been observed 48 h after radiation [28]. Similar results have been declared after evaluating changes in *CUGBP2* mRNA and protein expression under radiation in RC cell cultures, where an increase in CUGBP2 expression has been observed 24 h after radiotherapy [29]. It might be possible that changes in *CUGBP2* and *HUR* gene expression after radiotherapy can program further the fate of COX2 and VEGFA, for example, a fast disruption of mRNA, stabilization and the silencing of translation. Although CUGBP2 is known to stabilize *COX2* mRNA and inhibit translation to protein, the increase in *COX2* mRNA expression was insignificant, possibly confirming post-transcriptional gene expression regulation. 

A significant decrease in *VEGFA* mRNA expression after neoadjuvant therapy was detected in this study. It has previously been shown that radiotherapy has the ability to inhibit tumor angiogenesis [59] and this ability might be associated with the inhibition of *VEGFA* expression, leading to a decreased amount of *VEGFA* mRNA in tissues. Moreover, a stronger downregulation of the pro-oncogenic *VEGFA* and *COX2* mRNA following neoadjuvant therapy was observed in the group of patients with downstaged tumors (based on T-stage downstaging), while non-downstaged tumors exhibited higher *VEGFA* and *COX2* mRNA expression. Similarly, high levels of COX2 protein expression after RT or CRT have been previously associated with low levels of tumor cell apoptosis, poor prognosis or minor histopathologic response in patients with rectal or esophageal cancer [60,61]. As we know, an increased secretion of COX2 stimulates PGE2 production and therefore increases cell proliferation. PGE2 also increases cell immunity to radiation and promotes VEGFA expression, increasing the small vascular-net formation in tumors [44,62].

It is important to mention the limitations of this study, the main one being the small amount of suitable tissue for analysis. Due to RT-induced fibrosis, a large portion of the post-treatment tissue samples was unsuitable for further analysis. Therefore, a more extensive study in a larger group of patients is needed to verify the connection between the changes in *VEGFA*, *COX2*, *HUR* and *CUGBP2* gene expression under neoadjuvant therapy and the efficacy of the treatment.

## 5. Conclusions

In conclusion, we confirmed that *VEGFA*, *COX2* and *HUR* genes are overexpressed in RC tissue and pre-treatment *VEGFA* expression is higher in patients with tumor T-stage downstaging, highlighting their role in RC pathogenesis and predictive potential. Our study also shows that lower *VEGFA* and *COX2* expression levels after neoadjuvant therapy could indicate a better response to treatment.

## Figures and Tables

**Table 1:** Summary of clinical characteristics of rectal cancer (RC) patients. RT—radiotherapy, CRT—chemoradiotherapy, SD—standard deviation.

**Number**	27	
**Age** (Mean ± SD)	66.5 ± 8.75	
**Gender, *N* (%)**		
Male	18 (67)	
Female	9 (33)	
**Downstaging, *N* (%)**	*Based on T-stage (cT → ypT)*	*Based on T/N stages* (*cT/cN → ypT/ypN; AJCC staging cII-III → yp0-I*)
Downstaged	9 (33)	7 (26)
Non-downstaged	14 (52)	16 (59)
No data	4 (15)	4 (15)
**Neoadjuvant therapy, *N* (%)**		
RT	11 (41)	
CRT	16 (59)	

## Supplementary Materials

The following are available online at https://www.mdpi.com/1010-660X/56/4/192/s1, Table S1: A summary of patient distribution according to AJCC and TNM classification.
