# Supplementary material for: The Role of VEGFA, COX2, HUR and CUGBP2 in Predicting the Response to Neoadjuvant Therapy in Rectal Cancer Patients
Source: Medicina (Kaunas). 2020 Apr 22;56(4):192. doi: 10.3390/medicina56040192 (PMC7230171; doi:10.3390/medicina56040192)
Supplement: Supplementary file 1 [file medicina-56-00192-s001.pdf]

Table S1:. A summary of patient distribution according to AJCC and TNM classification.

**Before neoadjuvant therapy, N (%)**	**After neoadjuvant therapy, N (%)**
Stage **cIII** 22 (81.5):<br>cT4N+ 6 (22)<br>cT3N+ 13 (48)<br>cT2N+ 3 (11)<br><br>Stage **cII** 1 (3.5):<br>cT3N0 1 (3.5)<br><br>No data 4 (15)	Stage **ypIII** 8 (29.5):<br>ypT3N+ 7 (26)<br>ypT2N+ 1 (3.5)<br><br>Stage **ypII** 8 (29.5):<br>ypT3N0 8 (29.5)<br><br>Stage **ypI** 7 (26):<br>ypT2N0 7 (26)<br><br>No data 4 (15)
